# The association of sociodemographic factors and history of chronic diseases on menopausal symptoms: A cross‐sectional study

**DOI:** 10.1002/hsr2.2103

**Published:** 2024-05-06

**Authors:** Seyedeh Hajar Sharami, Zahra Rafiei Sorouri, Sara Farzadi, Fatemeh Hosseinzadeh, Atoosa Etezadi, Sedighe Bab Eghbal, Habib Eslami‐Kenarsari

**Affiliations:** ^1^ Department of Obstetrics and Gynecology, Reproductive Health Research Center, Al‐Zahra Hospital, School of Medicine Guilan University of Medical Sciences Rasht Iran; ^2^ Department of Obstetrics & Gynecology, Reproductive Health Research Center, Al‐Zahra Hospital, School of Medicine Guilan University of Medical Sciences Rasht Iran; ^3^ Vice‐Chancellorship of Research and Technology Guilan University of Medical Science Rasht Iran

**Keywords:** menopause, prevalence, risk factors, women

## Abstract

**Background:**

Menopausal symptoms are very diverse in terms of prevalence and severity, and this difference is due to various factors such as psychological factors, sociocultural status, lifestyle, geographical location, and other factors. This study aimed to assess the prevalence of menopausal symptoms and evaluate the predictive factors related to the prevalence and severity of menopausal symptoms.

**Materials and Methods:**

This was a cross‐sectional analytical study that was performed on 214 women aged 35–65 years old who were referred to Alzahra Educational, Research and Treatment Center in Rasht, Iran. The data collection tool was a valid and reliable questionnaire, using the list of menopausal symptoms and a checklist of subjects’ general characteristics.

**Results:**

16.8% of postmenopausal women in our study had at least one menopausal symptom. Using multiple linear regression, race (*p* = 0.02), history of chronic diseases (*p* = 0.04), place of residence (*p* = 0.02), and marital satisfaction (*p* = 0.02) were associated with menopausal symptoms. Nineteen percent of the covariates related to the logistics function were explained by the predictor variables in the model.

**Conclusion:**

Evaluation of menopausal symptoms showed that the severity of menopausal symptoms was related to factors such as body mass index (BMI), ethnicity, place of residence, marital satisfaction, and history of chronic diseases, and need to address BMI, psychological issues, and chronic illness.

## 1 BACKGROUND

Menopause is caused by ovarian function disruption, which means a woman's reproductive end life, and is accompanied by a decrease in the level steroid hormones ovarian. Menopause occurs for many women between the ages of 47 and 55. Perimenopause, the time of change in ovarian function, usually lasts 2–8 years (average 3.8 years). This period clinical manifestations to menopause are not well understood.^1^, ^2^

The aging population is on the rise, according to the World Health Organization.^3^, ^4^ During menopause, women often suffer from a variety of symptoms that may adversely affect their daily activities, physical and mental health, and quality of life.^5^ Numerous symptoms are manifested by changes in the hormonal status associated with menopause, and in most women, the direct relationship between menopause and nonspecific physical and mental symptoms such as hot flashes, vaginal dryness, sleep disturbance, and problems with concentration and or memory are observed. In addition, during menopause, women may experience symptoms of mood swings, and depression. Understanding risk factors, clinical manifestations, and managing common menopausal symptoms improves patient care and women's health outcomes.^6^

Different studies provide different information on the factors associated with menopausal symptoms. In some studies, education, socioeconomic level, number of deliveries, breastfeeding, and taking birth control pills^7^ and in others personal income, parity more than four, abortion, calcium supplementation, and a heart disease history and rheumatoid arthritis^8^ were considered as factors related to menopausal symptoms.

Due to the existing controversies, geographical differences, and the fact that the factors related to menopausal symptoms can change over time, this cross‐sectional study was performed to examine menopausal factors in women aged 35–65 years referred to Alzahra Educational, Research and Treatment Center in Rasht, Iran.

## 2 MATERIALS AND METHODS

### 2.1 Study setting

This analytical cross‐sectional study was performed on 214 women aged 35–65 years referred to Alzahra Educational, Research and Treatment Center from April 2020 to December 2020. The study sample size was calculated using 5% accuracy, 95% reliability coefficient, and previous similar study. Inclusion criteria were women aged 35–65 years old^9^ visiting primary care for gynecological and nongynecological reasons and exclusion criteria were undiagnosed vaginal bleeding–heart disease–liver disorder–complicated diabetes mellitus with optical, cardiac, and renal complications–breast tumor or other malignancies–stroke history or transient ischemic attack–hormone therapy history in the last 12 months^10^ women with medically or surgically induced menopause–pregnant or lactating women^11^ and unwillingness to participate in the study.

### 2.2 Instrument and data collection

The data collection tool was a researcher‐made questionnaire. The questionnaire was divided into 2 parts. Part 1: 27 questions related to demographic and fertility characteristics. The second part was 29 questions about menopausal symptoms (physical symptoms include 11 questions, psychological symptoms include 12 questions, and urinary–genital symptoms include 6 questions). For scoring the menopausal symptoms questionnaire, the score for each question was between 1‐4, so that the answer was never (never in the last 3 months): score 1, occasionally (5 times in the last 3 months): score 2, often (more than 10 times in the last 3 months): score 3, almost always (daily in the last 3 months): score 4 were given. Score 1 meant no problem and score 4 meant having a lot of problems.

To assess the validity of the questionnaire, the content validity method was used, so that the menopause symptoms questionnaire was prepared based on the list of menopausal symptoms^12^ and a checklist of subjects' general characteristics.

Considering the frequency scale of 56 menopausal symptoms in terms of evaluating the validity of translating the questionnaire in both source and target languages using the standard method (backward–forward) as a cross‐cultural adaptation guide, the questionnaire was conducted by the research team and a total of 29 questions Menopausal symptoms were obtained.

Forward method was used and The questionnaire was given to 14 who had sufficient expertise and experience and the content validity index (CVI) and content validity ratio (CVR) were calculated. The value of CVR of 27 questions was less than 0.57, which was removed and the CVI obtained in this study was 0.82. After the corrections, its validity was confirmed.

The reliability of the questionnaire was measured by Cronbach's *α* test on 30 women who were similar in demographic characteristics to the study population with a test value of 0.702 for physical symptoms, 0.916 for psychological symptoms, and urinary–genital symptoms was estimated at 0.731. The questionnaire was collected by the researcher in the form of face‐to‐face interviews after obtaining written consent. Each interview lasted 15–20 min.

It should be noted that 3 women who referred to the gynecology hospital refused to complete the questionnaire and Also, 30 people were deleted from the program during the interview due to the lack of inclusion criteria.

### 2.3 Statistical analysis

Data were analyzed using SPSS software version 21, using descriptive statistical methods for demographic information. To compare the frequency and symptoms severity Mann–Whitney and Kruskal–Wallis tests were used. To determine the factors associated with the severity of menopausal symptoms (somatic, psychological, and urogenital) multiple linear regression analysis was used.

## 3 RESULTS

The majority of participants were married (89.3%), their residence place was the city (65.4%), and their ethnicity was Gilak (70.1%), and had marital satisfaction (79.9%) (Table 1).

**Table 1:** Baseline characteristics of the study participants.

Characteristics	All (%) (*N *= 214)
Marital status	
Unmarried, divorced, or widowed	23 (10.7%)
Married	191 (89.3%)
Age	
<40	64 (29.9%)
40–50	81 (37.8%)
>50	69 (32.3%)
Weight (kg)	
$\bar{X}$ ± SD	75.59 ± 1.37
Height (cm)	
$\bar{X}$ ± SD	158.79 ± 5.68
Body mass index	
<25	32 (14.9%)
25–30	90 (42.1%)
>30	92 (43%)
Ethnicity	
Gilak	150 (70.1%)
Other	64 (29.9%)
Occupation	
Housewife	198 (92.5%)
Employed	16 (7.5%)
Education	
Less than diploma	139 (65%)
diploma and more	75 (35%)
Spouse's occupation	
Unemployed	75 (35%)
Employed	139 (65%)
Spouse's education	
Less than diploma	126 (65.3%)
Diploma and more	67 (34.7%)
Chronic disease history	
Yes	111 (51.9%)
No	103 (48.1%)
Residence place	
Urban	140 (65.4%)
Rural	74 (34.6%)
Marital satisfaction	
Single	7 (3.3%)
Yes	171 (79.9%)
No	36 (16.8%)

In participants over 50 years, menopausal symptoms score in somatic and urogenital domains were higher than other participants (*p* < 0.032). Women with normal body mass index (BMI) have a lower overall score compared to overweight or obese women, and only the somatic score is lower and statistically significant in women with normal BMI (*p* = 0.045). With the increasing of the spouse's education to diploma and more, the score of menopausal symptoms severity was lower in all domains and only menopausal symptoms severity was significant in the psychological domain (*p* = 0.046).

The highest prevalence of chronic history diseases was related to hyperlipidemia 40 (18.7%) while the lowest prevalence was related to fatty liver 1 (0.5%) and menopausal symptoms severity was significant in all domains and in general (*p* = 0.001).

Women who are dissatisfied with their married life have a higher score of menopausal symptoms severity in the somatic (22.53 ± 6.65), psychological (25.17 ± 9.08), and urogenital (12.97 ± 4.01) domains than those who are satisfied with their married life and not married participants, and these differences were statistically significant (*p* < 0.049) (Table 2).

**Table 2:** Menopausal symptoms scores in study participants.

	*N*	Somatic	Psychological	Urogenital	Total score
Marital status					
Unmarried, divorced, or widowed	23 (10.7%)	21 ± 6.28	23.74 ± 8.26	12.17 ± 3.08	56.91 ± 13.99
Married	191 (89.3%)	19.59 ± 6.08	21.90 ± 8.39	11.33 ± 4.28	52.82 ± 15.33
*p* Value	Mann–Whitney	0.255	0.274	0.220	0.138
Age					
<40	64 (29.9%)	18.11 ± 4.81	22.26 ± 8.58	10.83 ± 3.89	51.20 ± 14.40
40–50	81 (37.8%)	19.97 ± 6.77	22.51 ± 8.44	10.02 ± 4.01	52.50 ± 16.02
>50	69 (32.3%)	20.98 ± 6.10	21.46 ± 8.20	13.61 ± 3.74	56.06 ± 14.78
*p* Value	Kruskal–Wallis	0.032	0.687	0.001	0.132
BMI					
Below 25	32 (14.9%)	18.84 ± 5.54	20.09 ± 7.63	12.19 ± 4.06	51.12 ± 13.86
25–30	90 (42.1%)	18.89 ± 6.10	21.56 ± 8.03	11.44 ± 4.34	51.89 ± 15.18
Up to 30	92 (43%)	20.89 ± 6.17	23.33 ± 8.84	11.13 ± 4.05	55.35 ± 15.59
*p* Value	Kruskal–Wallis	0.045	0.139	0.425	0.174
Ethnicity					
Gilak	150 (70.1%)	19.76 ± 6.13	21.33 ± 7.63	11.27 ± 4.18	52.37 ± 14.55
Other	64 (29.9%)	19.70 ± 6.11	23.89 ± 9.74	11.76 ± 4.17	55.36 ± 16.59
p Value	Mann–Whitney	0.953	0.129	0.406	0.269
Occupation					
Housewife	198 (92.5%)	19.68 ± 6.09	22.04 ± 8.35	11.47 ± 4.20	53.20 ± 15.20
Employed	16 (7.5%)	20.50 ± 6.41	22.75 ± 8.93	10.75 ± 3.83	54.00 ± 15.89
*p* Value	Mann–Whitney	0.473	0.707	0.537	0.774
Education					
Less than diploma	139 (65%)	20.28 ± 6.43	22.58 ± 8.60	11.83 ± 4.31	54.69 ± 15.73
Diploma and more	75 (35%)	18.75 ± 5.37	21.20 ± 7.92	10.67 ± 3.81	50.61 ± 13.92
*p* Value	Mann–Whitney	0.181	0.321	0.078	0.096
Spouse's occupation					
Unemployed	75 (35%)	19.04 ± 5.74	21.76 ± 8.09	11.84 ± 4.16	52.64 ± 14.68
Employed	139 (65%)	20.12 ± 6.28	22.28 ± 8.55	11.19 ± 4.18	53.59 ± 15.54
*p* Value	Mann–Whitney	0.242	0.676	0.268	0.853
Spouse's education					
Less than diploma	126 (65.3%)	20.08 ± 6.36	22.61 ± 8.28	11.61 ± 4.39	54.30 ± 15.65
Diploma and more	67 (34.7%)	18.91 ± 5.64	20.67 ± 8.44	10.94 ± 4.09	50.52 ± 14.57
*p* Value	Mann–Whitney	0.307	0.046	0.330	0.122
Chronic disease history					
Yes	111 (51.9%)	20.79 ± 6.07	22.94 ± 7.80	12.42 ± 4.23	56.15 ± 14.59
No	103 (48.1%)	18.61 ± 5.98	21.19 ± 8.91	10.34 ± 3.79	50.14 ± 15.32
*p* Value	Mann–Whitney	0.004	0.009	0.001	0.001
Residence place					
Urban	140 (65.4%)	19.97 ± 6.13	22.76 ± 8.64	11.38 ± 4.16	54.12 ± 15.78
Rural	74 (34.6%)	19.31 ± 6.08	20.84 ± 7.75	11.48 ± 4.22	51.63 ± 14.04
*p* Value	Mann–Whitney	0.382	0.114	0.884	0.344
Marital satisfaction					
Single	7 (3.3%)	18.43 ± 5.79	20.71 ± 7.82	11.43 ± 3.59	50.57 ± 15.92
Yes	171 (79.9%)	19.21 ± 5.87	21.51 ± 8.15	11.09 ± 4.17	51.81 ± 14.72
No	36 (16.8%)	22.53 ± 6.65	25.17 ± 9.08	12.97 ± 4.01	60.67 ± 15.65
*p* Value	Kruskal–Wallis	0.016	0.049	0.042	0.007

Abbreviation: BMI, body mass index.

To determine the predictive power, multiple linear regression analysis showed a statistically significant relationship between menopausal symptoms and ethnicity, chronic diseases history, residence place, and marital satisfaction (*p* < 0.041) (Table 3).

**Table 3:** Multiple linear regression results for variables associated with menopausal symptoms.

Variables	Model of total		
	*β*	*t*	*p*
Ethnicity	0.170	2.306	0.022
Chronic disease history	0.153	2.059	0.041
Residence place	−0.178	−2.303	0.023
Marital satisfaction	0.168	2.264	0.025
Constant		6.064	<0.001
Adjusted *R* ^2^	0.194		
*F*	6.131		
*p*	<0.001		

## 4 DISCUSSION

In this study, the score of menopausal symptoms severity in the somatic and urogenital domains was higher, because, with increasing age, changes in somatic symptoms (movement speed, coordination, muscular endurance, balance) and changes in the urogenital system are observed, consistent with the study by Rindner et al. And preventive counseling for women before age 45 should be evaluated^13^ and in diseases presence, this weakness increases.

In the present study, only 34.7% of spouses had a high school diploma or more. When men are more educated, they have more knowledge, which prepares them to solve menopausal problems and helps them avoid conflict.^14^

Education may affect a person's behaviors and provides the ability to recognize and adapt to other people's feelings. Marital satisfaction means sense of pleasure, satisfaction, and joy that couples feel in all aspects of their lives. In addition, researchers believe that sex is the most important determinant of marital satisfaction.^15^ The study by Yarelahi et al. showed that couples marital satisfaction is affected by each health of them. Sexual problems in women can affect their husbands' marital satisfaction.^16^ Some studies have shown that marital satisfaction decreases in middle age due to increased health problems such as depression or financial stress.^14^ Women who enjoyed stronger family support, intimacy, and family relationships seemed to be less likely to have severe psychological symptoms.

The difference in BMI is a reliable indicator of hot flashes. In this study, 39.3% of women gained weight in the past year and only somatic symptoms score of people with BMI above 30 was higher than people with BMI between 25‐30 and also more than people with BMI less than 25 and these differences were statistically significant (*p* = 0.04). In this study, overweight or obese women were associated with increased severity of soamtic symptoms and with increasing BMI in women, menopausal symptoms such as tiredness and lack of energy, restlessness, and urinary incontinence increase, but the symptoms of poor appetite and vaginal dryness decrease. Similar to this study, in other studies, increased BMI, overweight, or obesity were associated with increased vasomotor intensity and somatic symptoms^17^ and more severe menopausal symptoms.^18^ Contrary to these results, it was found in Thapa and Yang study that there was no association between BMI and menopausal symptoms.^8^

There are two conflicting hypotheses about the relationship between BMI and menopausal symptoms. The “thin hypothesis” states that overweight women experience fewer vasomotor symptoms because cytochrome p450 aromatase in adipose tissue can convert androgens to estrogen.^19^ On the other hand, the “thermoregulatory model” expresses a positive relationship between BMI and vasomotor symptoms, which is due to the strong insulating adipose tissue capacity in the body^19^, ^20^ and prevents heat loss. Therefore, obese women may experience an increase in climacteric symptoms. Other studies have found a positive association between BMI and menopausal symptoms that support the thermoregulatory model.^20^, ^21^ Further prospective studies are needed to link obesity mechanism and menopausal symptoms.

In the present study, menopausal symptoms severity was associated with a history of chronic diseases such as hyperlipidemia (somatic and urogenital symptoms and in total) and diabetes (somatic symptoms and in total), and hypertension (urogenital symptoms) (*p* < 0.04) and women with a chronic disease history have 0.15 times more menopausal symptoms than women without a chronic disease history while in another study hypertension diseases, heart disease, depression, and rheumatoid arthritis were reported.^8^ In the study of Wang et al. chronic disease history was not a predictor^22^ and further studies are needed to link various chronic diseases history and menopausal symptoms.

Using multiple linear regression analysis, ethnicity, chronic diseases history, residence place, and marital satisfaction were among the predictor factors of menopause‐related symptoms. In our study, as in the study of Lan et al., the residence place has a significant relationship with the menopausal syndrome occurrence.^23^ Menopausal symptoms severity in women living in rural areas is 0.17 times lower than women living in urban areas. This may be due to the less stress, anxiety, life pressure, and no pollution with rural women air pollutants than urban women, which exposes them to fewer symptoms. Due to the predictive value of the aforementioned variables in menopausal symptoms severity, it is necessary to provide health services (preventive–counseling–educational) and medical (therapeutic) to improve women's health to reduce menopausal symptoms.

This study limitation was that our findings were based on a cross‐sectional study, to confirm the factors associated with menopausal symptoms there seems to be a need for studies on a larger scale and in different geographical areas.

## 5 CONCLUSION

Menopausal symptoms severity depends on factors such as BMI, ethnicity, residence place, marital satisfaction, chronic diseases history while considering BMI and psychological issues and chronic diseases which are changeable, is very important, diseases complications and women's health problems can be addressed by implementing educational and counseling programs, preventive measures, health care, and treatment programs.

## AUTHOR CONTRIBUTIONS

**Seyedeh Hajar Sharami**: Project administration; supervision. **Zahra Rafiei Sorouri**: Conceptualization; validation. **Sara Farzadi**: Conceptualization; visualization. **Fatemeh Hosseinzadeh**: Data curation; investigation. **Atoosa Etezadi**: Data curation; investigation. **Sedighe Bab Eghbal**: Writing—original draft; writing—review and editing. **Habib Eslami‐Kenarsari**: Formal analysis.

## CONFLICT OF INTEREST STATEMENT

The authors declare no conflict of interest.

## ETHICS STATEMENT

This study was approved by the Ethics Committee of Guilan University of Medical Sciences (Approval ID: IR.GUMS.REC.1399.395). Informed consents were taken from all participants before data collection.

## TRANSPARENCY STATEMENT

The lead author Sedighe Bab Eghbal affirms that this manuscript is an honest, accurate, and transparent account of the study being reported; that no important aspects of the study have been omitted; and that any discrepancies from the study as planned (and, if relevant, registered) have been explained.

## Data Availability

Not applicable.
